# Fabrication of CeO_2_/GCE for Electrochemical Sensing of Hydroquinone

**DOI:** 10.3390/bios12100846

**Published:** 2022-10-08

**Authors:** Archana Chaudhary, Mohd Quasim Khan, Rais Ahmad Khan, Ali Alsalme, Khursheed Ahmad, Haekyoung Kim

**Affiliations:** 1 Department of Chemistry, Medi-Caps University, AB Road, Pigdamber, Rau, Indore 453331, M.P., India; 2 Department of Chemistry, M.M.D.C, Moradabad, M.J.P. Rohilkhand University, Bareilly 244001, U.P., India; 3 Department of Chemistry, College of Science, King Saud University, Riyadh 11451, Saudi Arabia; 4 School of Materials Science and Engineering, Yeungnam University, Gyeongsan 38541, Korea

**Keywords:** CeO_2_ nanoparticles, hydroquinone, electrochemical sensor, differential pulse voltammetry

## Abstract

Hydroquinone is a widely used derivative of phenol which has a negative influence on human beings and the environment. The determination of the accurate amount of hydroquinone is of great importance. Recently, the fabrication of an electrochemical sensing device has received enormous attention. In this study, we reported on the facile synthesis of cerium dioxide (CeO_2_) nanoparticles (NPs). The CeO_2_ NPs were synthesized using cerium nitrate hexahydrate as a precursor. For determining the physicochemical properties of synthesized CeO_2_ NPs, various advanced techniques, viz., powder X-ray diffraction (PXRD), scanning electron microscopy (SEM), energy-dispersive X-ray spectroscopy (EDX), and X-ray photoelectron spectroscopy (XPS), were studied. Further, these synthesized CeO_2_ NPs were used for the modification of a glassy carbon electrode (CeO_2_/GCE), which was utilized for the sensing of hydroquinone (HQ). A decent detection limit of 0.9 µM with a sensitivity of 0.41 µA/µM cm^2^ was exhibited by the modified electrode (CeO_2_/GCE). The CeO_2_/GCE also exhibited good stability, selectivity, and repeatability towards the determination of HQ.

## 1. Introduction

Hydroquinone (benzene 1,4-diol, HQ) is a positional isomer of a phenolic compound and is known for its high toxicity to the ecological environment and humans, even if it is used at very low concentrations [1,2]. If recent reports from the United States Environmental Protection Agency (UPA) and the European Union (EU) are taken into account, HQ is harmful to the environment due to its lower rate of degradability in ecological environments and its biohazard nature [3,4]. HQ finds a variety of applications in bleaching creams, pesticides, medicines, cosmetics, secondary coloring materials, photography chemicals, and flavoring compounds [5,6,7,8]. Owing to the large industrial use of HQ, it is being released into the environment and aquatic systems; therefore, it becomes essential to detect the traces of HQ [7].

For determining and quantifying the concentration of HQ in a system, a number of analytical ways are being applied, viz., gas chromatography-mass spectrometry (GCMS), high-performance liquid chromatography (HPLC), capillary electrochromatography, chemiluminescence, the fluorimetric method, flow injection analysis, spectrophotometry, electrochemical methods, etc. [9,10,11,12,13,14,15]. However, gas chromatography and high-performance liquid chromatography involve costly, sophisticated instruments and skilled staff alongside the usage of extra-pure and large volumes of organic solvents; hence, they are inappropriate for routine field analysis. On the other hand, in the determination of HQ via the spectrophotometric technique, the presence of correlated compounds interferes in the result. Additionally, the reproducibility of the results remains a prime concern in the fluorescence-based methods.

On the contrary, merits such as cost effectiveness, easy handling, rapid response, stability, high sensitivity, high selectivity, a wider detection range, and a lower limit of detection (LOD) makes the electrochemical method a promising analytical approach for the detection of pollutants [1,16,17,18]. Unfortunately, if bare electrodes such as glassy carbon/platinum/gold/graphite electrodes are used for the quantification and determination of HQ, they show a poor response and, sometimes, even a redox peak is not observed. Therefore, it becomes significant to fabricate new electrode materials which are cost effective, easy to handle, stable, and may exhibit excellent electrocatalytic activity and selectivity for the HQ.

In recent years, researchers have made significant efforts to determine different pollutants electrochemically using chemically modified electrodes (CMEs), as CMEs are consistent, economic, give a rapid response, and consume low energy, particularly during the in situ determination of pollutants [19,20]. For the assembly of CMEs, it is vital to select a suitable modifier with enhanced electrocatalytic activity or excellent conductivity. The modification of GC, gold and platinum electrodes by nanoparticles/nanocomposites of transition metal oxides, their composites, and conducting polymers have emerged as a leading area of research during the last decade [20,21,22,23,24,25].

In 2020, Mobin and the research team at IIT Indore developed a novel HQ sensor using the N-rGO/SrZrO_3_ composite as an electrode material [1]. In 2019, Ahmad et al. [21] also reported the fabrication of an HQ and catechol sensor using SnO_2_ as the electrode material. In another work, Meskher et al. fabricated NiO/rGO/f-MWCNTs on a platinum (Pt) electrode for the determination of HQ and catechol simultaneously [26]. This indicated that electrode materials have a significant role in the development of electrochemical sensors [1].

Cerium dioxide (CeO_2_) is a rare-earth metal oxide which possesses excellent biocompatibility, oxygen vacancy formation, and natural abundance [27]. CeO_2_ has been widely used in a variety of applications such as catalysis, solar cells, electrochemical sensors, energy storage, batteries, light-emitting diodes, photodetectors, and photocatalysis [28,29,30,31,32,33,34]. Temerk et al. modified a GC paste electrode using doped CeO_2_ NPs and applied it for electrochemical sensing of uric acid in a biological fluid [35]. The interest in the ceria nanoparticles (CeO_2_ NPs) is due to the presence of Ce^3+^ ions and generated oxygen-vacancies. The Ce^3+^ ions on the surface act as sites for catalytic reactions and generated oxygen vacancies help in the transformation and migration of active species present in that system [36].

Herein, we report a “benign approach for the synthesis of CeO_2_ NPs for electrochemical sensing of HQ”. The fabricated HQ sensor exhibits excellent sensing performance in terms of detection limit, sensitivity, and repeatability. Moreover, the fabricated CeO_2_/GCE demonstrates excellent selectivity towards the sensing of HQ in the presence of various electroactive species.

## 2. Materials and Methods

### 2.1. Chemicals

The chemicals, reagents, and solvents used for the experiment, viz., cerium nitrate hexahydrate (Ce(NO_3_)_3_.6H_2_O), glucose, ascorbic acid, dopamine, uric acid, potassium hydroxide (KOH), phosphate-buffered saline (PBS) solutions, and hydroquinone, were purchased from Alfa Aesar, Loba, SRL, Fisher Scientific, Sigma, Merck, and TCI. All the chemicals were used as received without any further purification or treatment. The stock solution of a 0.1 M concentration of HQ was prepared and used for the electrochemical sensing measurement.

### 2.2. Synthesis of CeO_2_

**Scheme 1:**
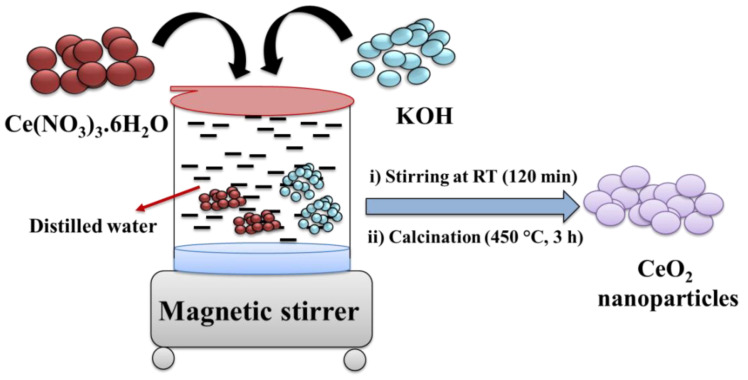
Schematic representation for the synthesis of CeO_2_ NPs.

The CeO_2_ NPs were prepared using the precipitation method, followed by calcination. In brief, we dissolved 3.26 g of Ce(NO_3_)_3_.6H_2_O in 25 mL of distilled water via stirring at room temperature (RT). After that, a 1 M solution of KOH was prepared using distilled water (D.W.). We slowly added the prepared 1M KOH solution to the aqueous solution of Ce(NO_3_)_3_.6H_2_O. The reaction mixture was stirred for 2 h at RT. Further, the precipitate was collected through centrifugation and dried at 60 °C overnight. Finally, it was calcinated at 450 °C for 3 h, which yielded CeO_2_ NPs (Scheme 1).

### 2.3. Fabrication of Electrode

**Scheme 2:**
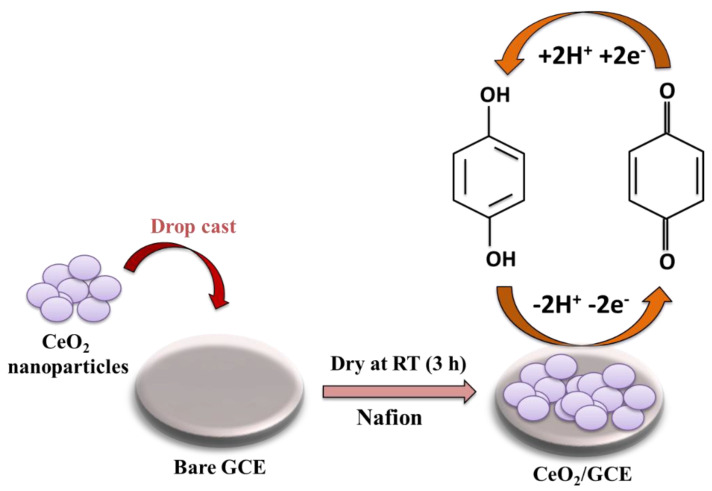
Schematic picture shows the fabrication and working of CeO_2_/GCE.

The bare GCE was cleaned with an alumina slurry (0.5 µm) and velvet pad. Further, 3.5 mg of CeO_2_ NPs was dispersed in 3 mL of DW (0.1 wt% Nafion) using ultrasonication for 1 h. The cleaned GCE (3 mm) was modified with 7.5 µL of CeO_2_ ink and dried at room temperature for 3 h (Scheme 2).

This CeO_2_-modified GCE is denoted as CeO_2_/GCE and used as the working electrode in this experiment. Here, the reference electrode is silver/silver chloride (Ag/AgCl), whereas the counter electrode is a platinum electrode (the Ag/AgCl electrode was filled with a 3 M KCl solution). We performed all the electrochemical measurements on three electrode computer-controlled potentiostat/galvanostat systems (CH Instruments).

## 3. Results

### 3.1. Physiochemical Properties of CeO_2_

**Figure 1:**
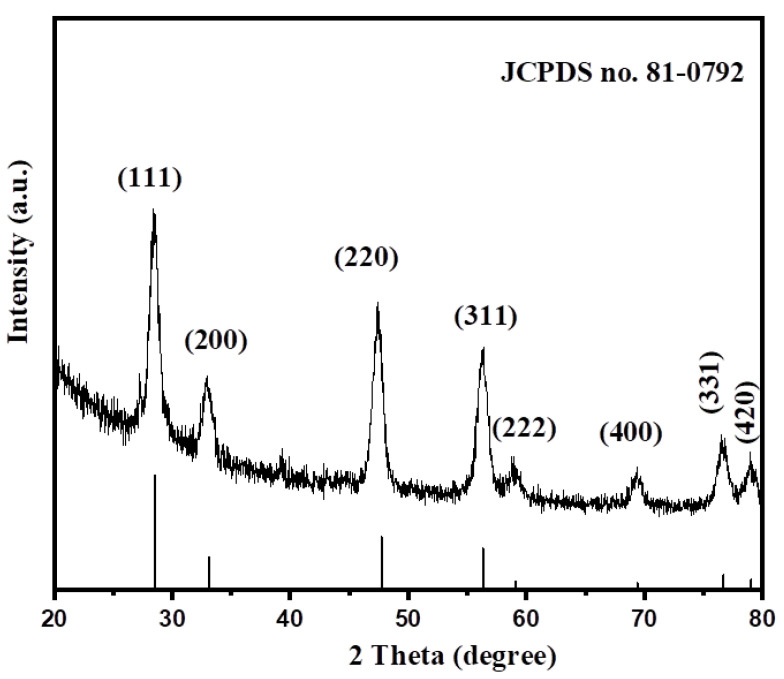
PXRD pattern of the synthesized CeO_2_.

We used the powder X-ray diffraction (PXRD, Rigaku) method to confirm the structural phase of CeO_2_. The PXRD pattern of the obtained CeO_2_ NPs between the 2θ range of 10–80° is presented in Figure 1. The diffraction peaks are observed at ~28.66°, 33.03°, 47.56°, 56.39°, 59.05°, 69.34°, 76.61°, and 79.27°, which relate to (111), (200), (220), (311), (222), (400), (331), and (420) planes. The observed reflections exhibit the formation of the face-centered cubic phase of CeO_2_ where the lattice parameters are a = b = c = 0.5412 nm. The obtained PXRD pattern of the CeO_2_ NPs was well-matched with the previously reported JCPDS file No: 81–0792. The absence of any other additional peaks in the PXRD reveals the phase purity of the CeO_2_ sample. Thus, it can be said that CeO_2_ NPs have been prepared with high-phase purity by the precipitation method.

The average crystallite size of the synthesized CeO_2_ has been calculated employing the Debye–Scherrer equation given below [37].

$$D\;=\frac{0.9\;\lambda }{\beta \;\cos\;\theta }$$

where D = Average crystallite size (Å), λ = X-ray wavelength (0.154 nm), θ = diffraction angle, β = full width at half maximum (FWHM) of the observed peak.

**Figure 2:**
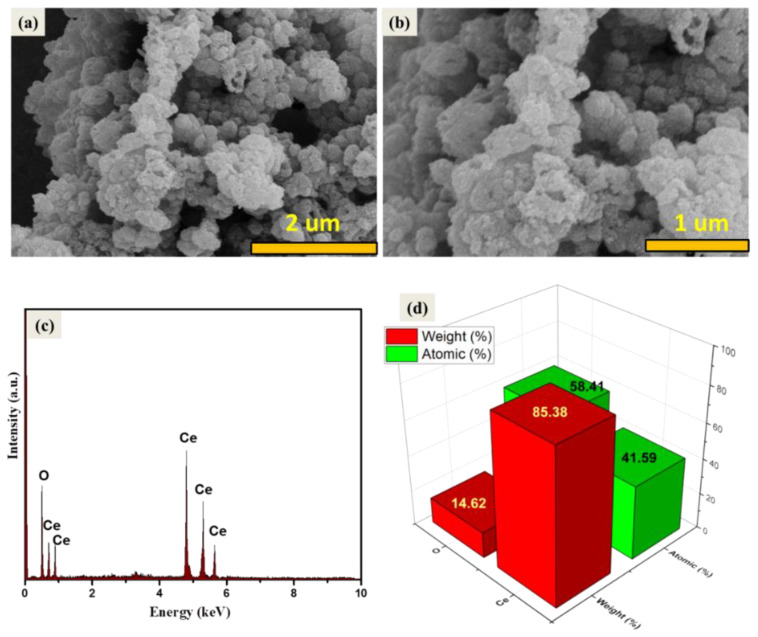
SEM images (**a**,**b**), EDX spectrum (**c**), and elemental composition (**d**) of the synthesized CeO_2_ NPs.

The average crystallite size of the prepared CeO_2_ NPs was found to be 23.83 nm. Our results related to the crystallographic properties of the prepared CeO_2_ are in accordance with the previously reported literature [37]. The surface morphological/structural properties play a significant role in the development of electrochemical and optoelectronic applications. Therefore, it is required to examine the morphological features of the prepared CeO_2_ NPs. Thus, the surface morphology of the prepared CeO_2_ sample has been investigated using scanning electron microscopy (SEM, Zeiss). The recorded microscopic SEM images of the CeO_2_ sample are displayed in Figure 2a,b. The SEM results indicated the presence of an agglomeration of the CeO_2_ NPs. Furthermore, we have also examined the phase purity of the prepared CeO_2_ NPs using energy-dispersive X-ray spectroscopy (EDX). The EDX spectrum of the prepared CeO_2_ NPs was collected on Horiba EDX Instruments connected with the SEM instrument. The collected EDX spectrum of the Horiba EDX Instruments has been presented in Figure 2c. The EDX results indicated the presence of Ce and O elements in the prepared CeO_2_ NPs (Figure 2c). The atomic and weight percentage of the Ce and O elements in the CeO_2_ NPs are presented in Figure 2d. The EDX results confirmed the formation of CeO_2_ NPs with good purity.

**Figure 3:**
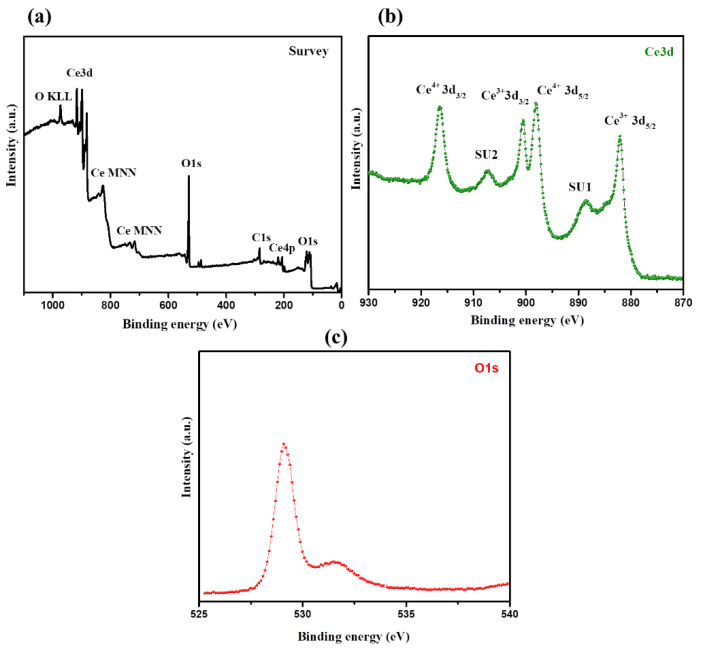
XPS of CeO_2_. Survey scan (**a**), Ce3d (**b**), and O1s (**c**) of the synthesized CeO_2_ NPs.

Furthermore, surface atomic compositions and valence states of the prepared CeO_2_ NPs have been studied via XPS analysis. According to the survey spectrum, CeO_2_ NPs contain only cerium, oxygen, and carbon elements (Figure 3a). The Ce 3D spectrum shows the peaks for both Ce^4+^ and Ce^3+^ species, revealing the reduction of Ce^4+^ to Ce^3+^. Under the conditions of high temperatures and low oxygen partial pressures, Ce^4+^ is partially reduced to Ce^3+^ [38]^.^ The XPS spectrum of the O1s region generally reveals knowledge about absorbed oxygen, oxygen vacancies, lattice oxygen, and the surface hydroxyl group (OH^−^) [36,38]. On the other side, the O1s spectrum shows asymmetric peaks for the CeO_2_ sample. Lower binding energy peaks (~528.6–528.8 eV) could be ascribed to O^2−^ ions associated with the lattice oxygen in the cubic structure, whereas a higher binding energy peak (~530.9 eV) could be ascribed to the O^2−^ ions present in the oxygen-deficient region [39]. The above studies confirmed the successful formation of CeO_2_ NPs with good phase purity [39].

### 3.2. Electrochemical Sensing Properties of CeO_2_/GCE

Initially, electrocatalytic activities of the GCE and CeO_2_/GCE were studied in a 5 mM [Fe(CN)_6_]^3−/4−^ redox solution. The cyclic voltammetry (CV) was used to obtained the CV graph of the GCE and CeO_2_/GCE in a 5 mM [Fe(CN)_6_]^3−/4−^ redox solution (scan rate = 50 mV/s). The obtained CV graphs of the GCE and CeO_2_/GCE in the 5 mM [Fe(CN)_6_]^3−/4−^ redox solution are presented in Figure S1a. The CV results exhibited the presence of a higher electrocatalytic current response for CeO_2_/GCE compared to the bare GCE (Figure S1a). Furthermore, electrochemical impedance spectroscopy (EIS) was also used to examine the electrocatalytic behavior of GCE and CeO_2_/GCE in the 5 mM [Fe(CN)_6_]^3−/4−^ redox solution. Figure S1b demonstrates the Nyquist plot of the GCE and CeO_2_/GCE in the 5 mM [Fe(CN)_6_]^3−/4−^ redox solution. The observations revealed that the bare GCE showed a large semicircle, which demonstrates the presence of a high charge-transfer resistance (R_ct_) of 833.4 Ω, whereas CeO_2_/GCE showed a small semicircle with an R_ct_ of 512.3 Ω. Thus, it can be said that CeO_2_/GCE has better electrocatalytic activity, which may be due to the presence of CeO_2_ NPs.

**Figure 4:**
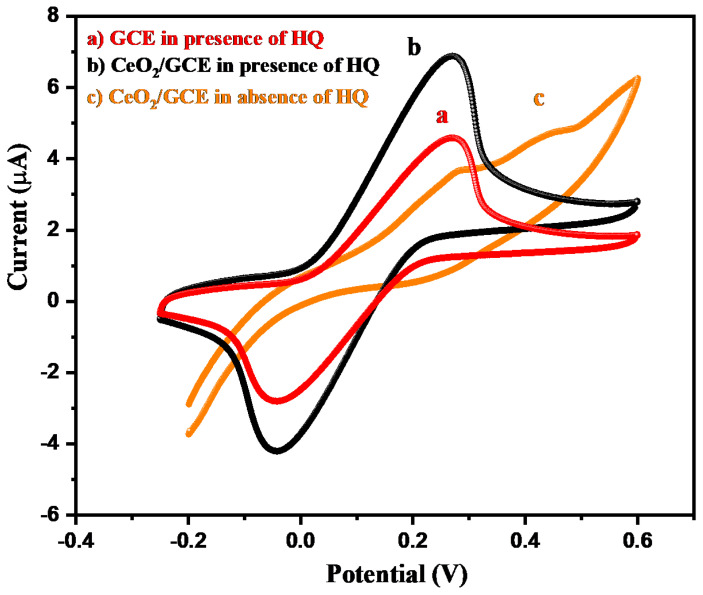
CV graph of GCE (**a**) and CeO_2_/GCE in presence (**b**) and absence (**c**) of 15 µM HQ in 0.1 M PBS of pH 7.0 (applied scan rate = 50 mV/s).

**Figure 5:**
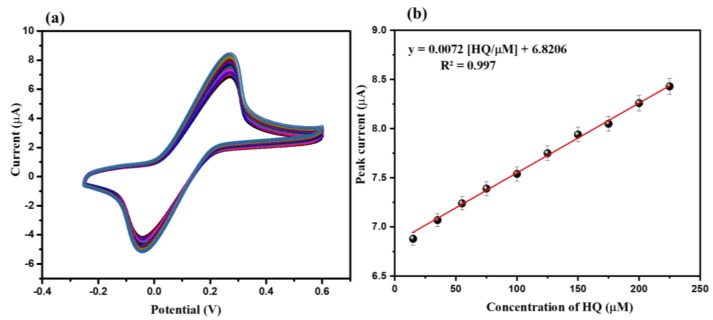
CVs (**a**) of CeO_2_/GCE in presence of HQ (15 µM, 35 µM, 55 µM, 75 µM, 100 µM, 125 µM, 150 µM, 175 µM, 200 µM, and 225 µM) in 0.1 M PBS (pH = 7.0; applied scan rate = 50 mV/s). Calibration plot (**b**) of peak current versus concentration.

The electrochemical sensing behavior of the CeO_2_/GCE and bare GCE were examined using the CV technique. The CV graphs of the CeO_2_/GCE and bare GCE were collected in the presence of 15 µM of HQ in 0.1 M PBS (pH of the PBS was 7.0) at the applied scan rate of 50 mV/s. Figure 4 demonstrates the collected CV graphs of the CeO_2_/GCE and bare GCE in the presence of 15 µM of HQ in 0.1 M PBS (pH = 7.0) at the applied scan rate of 50 mV/s in the potential range from −0.2 to 0.6 V. The bare GCE exhibits the oxidation peak at 0.27 V with an electrocatalytic current response of 4.65 µA (Figure 4). On the other hand, CeO_2_/GCE exhibits the oxidation peak at 0.27 V with an enhanced electrocatalytic current response of 7.01 µA (Figure 4). This indicated the successful surface modification of GCE with CeO_2_ NPs. This improved current response for CeO_2_/GCE was attributed to the presence of good electrochemical properties of CeO_2_ NPs. The CV of the CeO_2_/GCE was also obtained in the absence of HQ, which does not showed any peaks related to the HQ (Figure 4). Thus, it can be understood that CeO_2_/GCE can be further used as a potential electrochemical sensor for the detection of HQ. It is well-known that the concentration of the HQ can influence the electrochemical performance of the fabricated electrochemical sensor. Thus, we have further investigated the effect of various concentrations of the HQ on the sensing ability of the modified CeO_2_/GCE using the CV technique. The CV graphs of the CeO_2_/GCE were collected at various concentrations of HQ from 15 to 225 µM in 0.1 M PBS (pH = 7.0) at a fixed applied scan rate of 50 mV/s. Figure 5a showed the collected CV graphs of the CeO_2_/GCE at various concentrations of HQ from 15 to 225 µM in 0.1 M PBS (pH = 7.0) at a fixed applied scan rate of 50 mV/s. The observations indicated that the current response for CeO_2_/GCE increases with the increasing concentration of the HQ from 15 to 225 µM at a fixed scan rate of 50 mV/s (Figure 5a). The good sensing response of CeO_2_/GCE may be attributed to Ce^3+^ ions [40].

The calibration plot between the current responses against the concentrations of the HQ has been plotted. The calibration curve of the current response versus the concentration of the HQ has been presented in Figure 5b. The calibration plot suggests that the current response was increased linearly, where R^2^ = 0.99 (Figure 5b).

**Figure 6:**
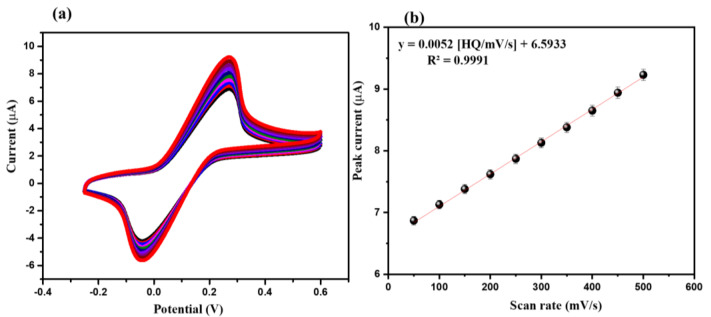
CVs (**a**) of CeO_2_/GCE in presence of 15 µM HQ in 0.1 M PBS (pH = 7.0) at different applied scan rates (50, 100, 150, 200, 250, 300, 350, 400, 450, and 500 mV/s). Calibration curve of peak current versus scan rate (**b**).

The influence of various applied scan rates on the electrochemical performance/ability of the CeO_2_/GCE was also checked using the CV technique. The CV graphs of the CeO_2_/GCE were obtained at a fixed concentration of 15 µM of HQ in 0.1 M PBS (pH = 7.0) at various applying scan rates (50 to 500 mV/s). The collected CV graphs of the CeO_2_/GCE in 15 µM of HQ in 0.1 M PBS at a pH of 7.0 are presented in Figure 6a. The CV results indicated that the current response for CeO_2_/GCE increases when applying scan rate changes from 50 mV/s to 500 mV/s (Figure 6a). The calibration curve between current responses and the square root of the applied scan rates was plotted, which is displayed in Figure 6b. The calibration plot between the current response and square root of the scan rate suggested that the current response increases linearly with an increasing applied scan rate, where R^2^ = 0.99 (Figure 6b).

**Figure 7:**
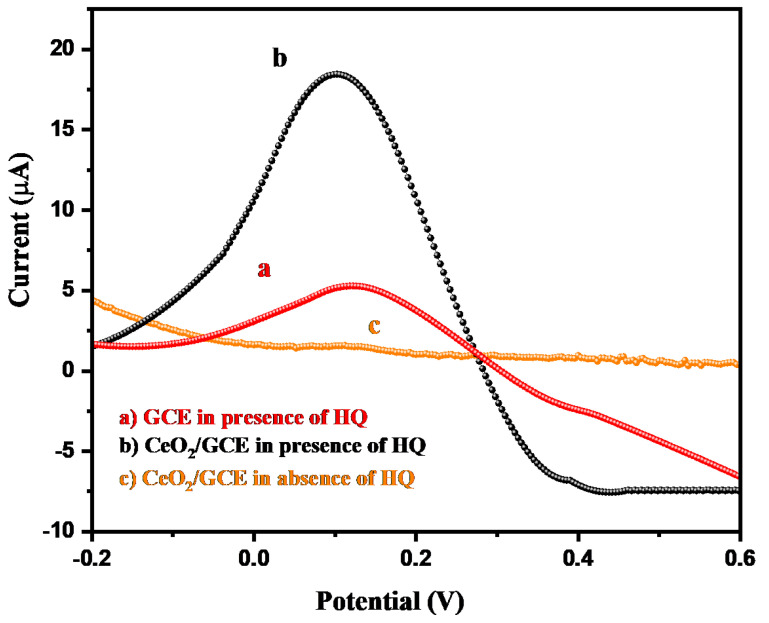
DPV graph of GCE (**a**) and CeO_2_/GCE in presence (**b**) and absence (**c**) of 15 µM HQ in 0.1 M PBS of pH 7.0 (applied scan rate = 50 mV/s).

According to the recently published literature, it has been observed that differential pulse voltammetry (DPV) is a highly sensitive and efficient electrochemical sensing technique compared to the CV or linear sweep voltammetry. Thus, we have also applied the DPV technique to detect the HQ using CeO_2_/GCE as the electrochemical sensor. The DPV graph of the CeO_2_/GCE and bare GCE were obtained in the presence of 15 µM of HQ in 0.1 M PBS (pH = 7.0) at the applied scan rate of 50 mV/s. The collected DPV graphs of the CeO_2_/GCE and bare GCE have been displayed in Figure 7.

The bare GCE demonstrates the current response of 5.57 µA towards the detection of 15 µM of HQ in 0.1 M PBS (pH = 7.0, scan rate = 50 mV/s). On the other side, CeO_2_/GCE exhibits significant improvement in the current response for the detection of 15 µM of HQ in 0.1 M PBS (pH = 7.0, scan rate = 50 mV/s). The highest DPV current response of 18.67 µA was obtained for CeO_2_/GCE, which is higher than that of the bare GCE (Figure 7). This clearly revealed that CeO_2_/GCE has better electrochemical sensing properties for the detection of HQ compared to the bare GCE using the DPV technique. The obtained DPV current response was found to be much better compared to the CV technique. Therefore, we have used the DPV technique for further electrochemical sensing measurements using CeO_2_/GCE as the HQ sensor.

**Figure 8:**
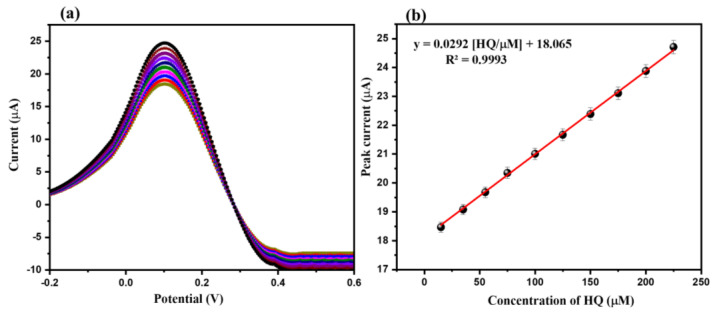
DPVs (**a**) of CeO_2_/GCE in presence of HQ (15, 35, 55, 75, 100, 125, 150, 175, 200, and 225 µM) in 0.1 M PBS (pH = 7.0) (applied scan rate = 50 mV/s). Calibration curve of peak current versus concentration (**b**).

The influence of the different concentrations of the HQ (15 µM, 35 µM, 55 µM, 75 µM, 100 µM, 125 µM, 150 µM, 175 µM, 200 µM, and 225 µM) on the electrochemical ability of the CeO_2_/GCE was also investigated. The DPV graphs of the CeO_2_/GCE were collected for various concentrations of HQ (15 µM, 35 µM, 55 µM, 75 µM, 100 µM, 125 µM, 150 µM, 175 µM, 200 µM, and 225 µM) in 0.1 M PBS (pH = 7.0) at a fixed scan rate of 50 mV/s. Figure 8a showed the DPV graphs of the CeO_2_/GCE in various concentrations of HQ (15 µM, 35 µM, 55 µM, 75 µM, 100 µM, 125 µM, 150 µM, 175 µM, 200 µM, and 225 µM) in 0.1 M PBS (pH = 7.0) at a fixed scan rate of 50 mV/s. The DPV results showed that the current response increases when increasing the concentration of HQ from 15 to 225 µM (Figure 8a). The calibration curve between the current response and concentration of HQ is presented in Figure 8b. The calibration plot between the current response and concentration of HQ suggests that the current response increases linearly, where R^2^ = 0.99.

The plausible sensing mechanism for the sensing of HQ by GCE/CeO_2_ has been drawn based on the previous literature [41]. The CV investigations suggest the involvement of the reversible oxidation–reduction reaction during the electrochemical detection of HQ.

Initially, oxygen–hydrogen bonds of both the phenolic -OH break, and subsequently, HQ is converted to quinoid by the release of two electrons and two protons (Scheme 2). Hence, it can be concluded that in the first step, the HQ is converted to quinone by liberating two protons that are later reverted to HQ and accept the liberated protons. The schematic representation is depicted in Scheme 2.

**Figure 9:**
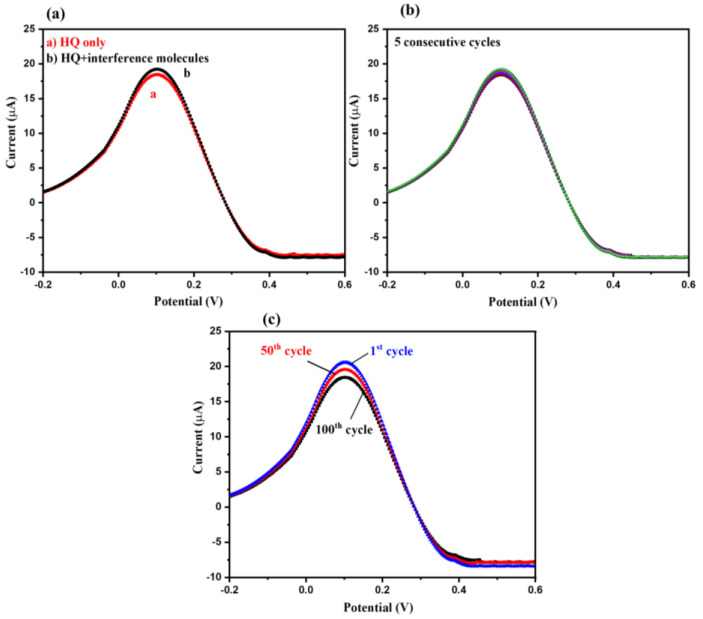
DPVs of CeO_2_/GCE. (**a**) Selectivity test: DPVs in presence of 15 µM HQ with (black) and without (red) interfering molecules (glucose, dopamine, H_2_O_2_, urea, uric acid, chlorophenol, nitrophenol, acetone, methanol, ascorbic acid, and hydrazine) at scan rate of 50 mV/s (concentration of interfering molecules was 5 times higher than that of HQ). (**b**) Repeatability test: 5 consecutive cycles of DPVs in presence of 15 µM HQ (scan rate = 50 mV/s). (**c**) Stability test: 1st, 50th, and 100th DPV cycle in 75 µM HQ (applied scan rate = 50 mV/s).

For selecting an ideal and efficient sensor, selectivity remains one of the most important parameters. Various interfering species create an interfering atmosphere, causing improper and inaccurate results during the detection of the desired analyte. Thus, in order to check the selectivity of CeO_2_/GCE for HQ sensing, we have applied the DPV method. First, the DPV curve of CeO_2_/GCE was recorded in the presence of 15 µM of HQ (Figure 9a); after that, the DPV curve of CeO_2_/GCE was recorded in the presence of 15 µM of HQ + various interfering molecules (glucose, dopamine, H_2_O_2_, urea, uric acid, resorcinol, chlorophenol, nitrophenol, acetone, methanol, ascorbic acid, and hydrazine). The concentration of the interfering species was five times higher than HQ. The DPVs were recorded at a scan rate of 50 mV/s. Insignificant variations in the current response were observed, as suggested by the obtained DPV curves, indicating the higher selectivity of CeO_2_/GCE against HQ. For the practical application of any sensor, its repeatability and stability are also to be taken into consideration. The repeatability of the designed CeO_2_/GCE electrode for sensing of HQ was also investigated by recording five consecutive DPV curves. DPV curves were recorded in the presence of 15 µM of HQ at a scan rate of 50 mV/s. The obtained DPVs exhibited good repeatability of CeO_2_/GCE for HQ sensing. In further studies, 100 DPV cycles of CeO_2_/GCE were obtained in 75 µM of HQ at a scan rate of 50 mV/s. The DPVs have been displayed in Figure 9c and observations revealed that CeO_2_/GCE has excellent stability up to 100 cycles.

The electrochemical sensing performance of any sensor can be evaluated by calculating the limit of detection (LoD) and sensitivity.

The LoD and sensitivity of the CeO_2_/GCE for HQ sensing were determined by using the following equations given below:

LoD = 3 × (σ_b_/S) (1)

where σ_b_ = standard deviation or error of blank, and S = slope of the calibration curve.

Sensitivity = S/A (2)

A = area of the working electrode.

The LoD of 0.9 µM and sensitivity of 0.41 µA µM^−1^cm^−2^ were obtained for CeO_2_/GCE, which is presented in Table 1.

In the past few years, a large number of HQ sensors have been reported which demonstrated good sensing performance. Through this connection, carbon spherical shells based on an HQ sensor were reported by Martoni et al. [42]. In another report, Maciel et al. [43] prepared poly (butylene adipate-co-terephthalate)/graphite as an HQ sensor. This fabricated sensor (poly (butylene adipate-co-terephthalate)/graphite) exhibited a decent LoD of 1.04 µM and sensitivity of 0.07 µA µM^−1^cm^−2^. In 2017, Zhang et al. [44] used a polarized GCE as the HQ sensor. Authors used the CV approach for the determination of HQ and obtained an LoD of 3.57 µM with a sensitivity of µA µM^−1^cm^−2^ [44]. Feng et al. [45] designed and synthesized carboxylic functional multi-walled carbon nanotubes using a layer-by-layer covalent-self-assembly approach. Further, the HQ sensor was developed using carboxylic functional multi-walled carbon nanotubes as the electrode modifier [45]. Authors modified the GCE with carboxylic functional multi-walled carbon nanotubes and investigated its sensing ability towards HQ detection using CV and DPV techniques [45]. The fabricated carboxylic functional multi-walled carbon nanotubes/GCE exhibited an LoD of 2.3 µM [45]. In another work, Hu et Al. [46] fabricated the rGO/MWCNTs composite and modified the GCE with rGO/MWCNTs as the electrode modifier. This modified GCE (rGO/MWCNTs/GCE) showed an LoD of 2.6 µM with excellent selectivity using CV and DPV techniques [46]. Umasankar et al. [47] prepared MWCNT-poly-malachite green on a GCE using the potentiodynamic method. The fabricated MWCNT-poly-malachite green/GCE was employed as the HQ sensor, which demonstrated an LoD of 1.6 µM. In another work, Erogul et al. [48] obtained an iron oxide (Fe_3_O_4_)-functionalized graphene oxide-gold nanoparticle (AuNPs/Fe_3_O_4_/APTESGO/GCE) and applied it as the HQ sensor. This fabricated electrode (AuNPs/Fe_3_O_4_/APTESGO/GCE) exhibits an LoD of 1.1 µM. In another work, a CNT/GCE-based HQ sensor exhibited an LoD of 2.9 µM [49], whereas Li et al. [50] fabricated **a** Zn/Al-layered double-hydroxide (LDHf) film on the GCE (LDHf/GCE). This fabricated LDHf/GCE showed an LoD of 9 µM [50]. Peng et al. [51] developed a GCE-based HQ sensor. This developed sensor showed the LoD of 8 µM [51]. Our obtained results are comparable with previously published sensors in terms of the LoD, as shown in Table 1 [42,43,44,45,46,47,48,49,50,51].

## 4. Conclusions

In conclusion, we can summarize that CeO_2_ NPs have been synthesized at room temperature via the precipitation method. The physical and chemical features of the synthesized CeO_2_ NPs were examined by using advanced characterization tools. Further, a hydroquinone sensor was developed using a glassy carbon electrode as the working substrate. The as obtained CeO_2_ NPs were drop-casted on to the active carbon surface of the glassy carbon electrode. The fabricated CeO_2_ NP-based glassy carbon electrode (CeO_2_/GCE) exhibits a reasonable limit of detection of 0.9 µM. The fabricated CeO_2_/GCE also showed a decent sensitivity of 0.41 µA µM^−1^cm^−2^. These observations indicated that CeO_2_/GCE has good electrocatalytic properties towards the sensing of hydroquinone. In further investigations, it was also found that CeO_2_/GCE possesses good repeatability, stability, and selectivity for the determination of hydroquinone using differential pulse voltammetry. We believe that the fabricated CeO_2_/GCE may be employed as a potential sensor for the determination of hydroquinone.

## Figures and Tables

**Table 1:** Comparison of LoD of CeO_2_/GCE with previous reports [42,43,44,45,46,47,48,49,50,51].

Material	LoD (µM)	Sensitivity (µA µM^−1^ cm^−2^)	References
CeO_2_/GCE	0.9	0.41	Present study
Carbon spherical shells	2.7	0.07	42
poly (butylene adipate-co-terephthalate)/graphite	1.04	-	43
Polarized GCE	3.57	0.136	44
Carboxylic functional multi-walled carbon nanotubes	2.3	-	45
rGO/MWNTs/GCE	2.6	-	46
MWCNT–poly-malachite green/GCE	1.6	-	47
AuNPs/Fe_3_O_4_/APTESGO/GCE	1.1	-	48
CNTs/GCE	2.9	-	49
LDHf/GCE	9	-	50
GCE	8	-	51

## Data Availability

Not applicable.

## Supplementary Materials

The following supporting information can be downloaded at: https://www.mdpi.com/article/10.3390/bios12100846/s1, Figure S1: CVs (a) and Nyquist plot (b) of GCE and CeO_2_/GCE in 5 mM [Fe(CN)_6_]^3−/4−^ and 0.1 M KCl in the applied frequency ranges of 0.1 Hz–100 kHz.
