# Supplementary material for: Fabrication of CeO2/GCE for Electrochemical Sensing of Hydroquinone
Source: Biosensors (Basel). 2022 Oct 8;12(10):846. doi: 10.3390/bios12100846 (PMC9599135; doi:10.3390/bios12100846)
Supplement: Supplementary file 1 [file biosensors-12-00846-s001.zip › biosensors-1931644-supplementary.pdf]

Supplementary Information: Fabrication of CeO_2/GCE for electrochemical sensing of hydroquinone

Archana Chaudhary[a,1,], Mohd Quasim Khan[b], Rais Ahmad Khan[c], Ali Alsalme[c,*], Khursheed Ahmad[d,1,], Haekyoung Kim[d,*]

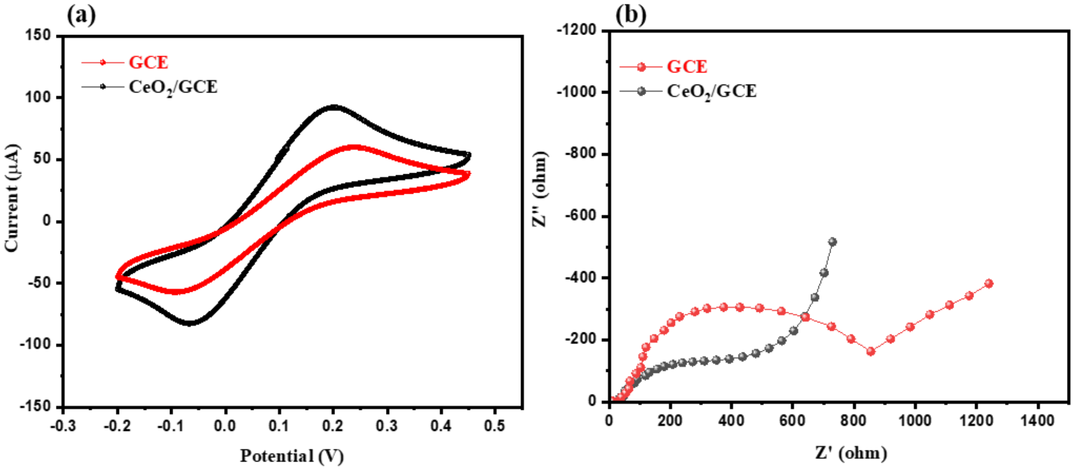

Figure S1. CVs (**a**) and Nyquist plot (**b**) of GCE and CeO_2/GCE in 5 mM $[Fe(CN)_6]^{3-/4-}$ in 0.1 M KCl in the applied frequency ranges of 0.1 Hz-100 kHz. .
